# Suitable temperature indicator for adverse health impacts in sub-tropical cities: a case study in Hong Kong from 2010-2019

**DOI:** 10.1007/s00484-024-02807-1

**Published:** 2024-10-30

**Authors:** Janice Ying-en Ho, Yitong Guo, Ka Chun Chong, Pak Wai Chan, Chun Kit Ho, Hiu Fai Law, Ren Chao, Edward Yan Yung Ng, Kevin Lau

**Affiliations:** 1 The Jockey Club School of Public Health and Primary Care, The Chinese University of Hong Kong, Hong Kong SAR, China; 2 Division of Landscape Architecture, Department of Architecture, Faculty of Architecture, The University of Hong Kong, Hong Kong SAR, China; 3 Hong Kong Observatory, Hong Kong SAR, China; 4 Institute of Future Cities, The Chinese University of Hong Kong, Hong Kong SAR, China; 5 School of Architecture, The Chinese University of Hong Kong, Hong Kong SAR, China; 6 Department of Civil, Environmental and Natural Resources Engineering, Luleå University of Technology, Room T3054, Luleå, 971 87 Sweden

**Keywords:** Night-time temperature, Heat-health, Climate change, Extreme heat, Temperature threshold, Early warning system

## Abstract

Heat-health warning systems and services are important preventive actions for extreme heat, however, global evidence differs on which temperature indicator is more informative for heat-health outcomes. We comprehensively assessed temperature predictors on their summer associations with adverse health impacts in a high-density subtropical city. Maximum, mean, and minimum temperatures were examined on their associations with non-cancer mortality and hospital admissions in Hong Kong during summer seasons 2010–2019 using Generalized Additive Models and Distributed Lag Non-linear Models. In summary, mean and minimum temperatures were identified as strong indicators for mortality, with a relative risk(RR) and 95% confidence interval(CI) of 1.037 (1.006–1.069) and 1.055 (1.019–1.092), respectively, at 95th percentile vs. optimal temperature. Additionally, minimum temperatures captured the effects of hospital admissions, RR1.009 (95%CI: 1.000- 1.018). In stratified analyses, significant associations were found for older adults, female sex, and respiratory-related outcomes. For comparison, there was no association between maximum temperature and health outcomes. With climate change and projected increase of night-time warming, the findings from this comprehensive assessment method are useful to strengthen heat prevention strategies and enhance heat-health warning systems. Other locations could refer to this comprehensive method to evaluate their heat risk, especially in highly urbanized environments and subtropical cities.

**Supplementary Information:**

The online version contains supplementary material available at 10.1007/s00484-024-02807-1.

## Introduction

The global mean temperatures of the 2010–2019 has made it the hottest decade on record thus far (World Meteorological Organization 2020). With climate change, extreme hot weather is projected to increase in intensity, severity, and duration in the foreseeable future (Intergovernmental Panel on Climate Change (IPCC) 2021). In Hong Kong, against the background of climate change and local urbanization, a significant increase of very hot days (daily maximum temperature ≥ 33 °C) and hot nights (daily minimum temperature ≥ 28^o^C) has been observed over the past decades (Hong Kong Observatory 2023). According to climate projections, the average annual number of very hot days and hot nights will further increase from 14 to 21 during 1995–2014 to 152 and 167 by 2081–2100 under a very high emissions scenario (SSP5-8.5) (Hong Kong Observatory 2021b). Extreme hot weather has been associated with adverse health impacts globally (Astrom et al. 2011; Basu 2009; Gasparrini et al. 2015; Gosling et al. 2009; World Meteorological Organization (WMO) & World Health Organization (WHO) 2015). Among various preventative actions, heat-health warning systems are essential to forecast, alert, and activate protective actions for the population.

Heat-health warning systems are triggered when the temperature exposure has exceeded a predefined threshold that is associated with adverse health outcomes (World Meteorological Organization (WMO) & World Health Organization (WHO) 2015). As there is no universal heat-health indicator for extreme heat (World Meteorological Organization (WMO) & World Health Organization (WHO) 2015), a comprehensive assessment is necessary to find a suitable indicator to fit the heat-health warning system according to the local climate and population health situation. Globally, previous studies compared which temperature measures could better predict health outcomes, but found little agreement among temperature predictors (Barnett et al. 2010; Kim et al. 2011; Schaeffer et al. 2016; Vaneckova et al. 2011), with some studies determining that none of the measures were superior (Barnett et al. 2010; Kim et al. 2011; Schaeffer et al. 2016). Two US studies found large variability between regions and neighbouring cities on the best performing indicator (Barnett et al. 2010; Davis et al. 2016). These findings demonstrate that location and local acclimatization may have significant impacts on heat-health effects, making local assessments and the development of city-specific models necessary (Davis et al. 2016).

A comprehensive assessment may be valuable to see the full influence of different temperature indicators. Previous studies mainly compared temperature indicators among mortality outcomes only, whereas other health outcomes such as hospitalization may have different patterns. A study in Arizona identified different thresholds on mortality, hospital admission, and emergency department visit outcomes, but did not assess model performance to differentiate between temperature indicators (Petitti et al. 2016). Additionally, the effect of indicators may vary between different age groups and disease groups. Among two studies conducted in Brisbane, Australia, Vaneckova et al. (2011) found no superiority among the temperature indicators, while Yu et al. (2011) suggested mean temperature was better for overall mortality, but some age groups and mortality categories (respiratory) were more sensitive to minimum temperatures.

In the highly-dense subtropical Hong Kong, previous studies have often used mean temperatures to assess mortality (Chan et al. 2012; Goggins et al. 2012; Ho et al. 2023) and hospitalization outcomes (Chan et al. 2013, 2018; Lam et al. 2016, 2018), and physical activity (Ho et al. 2022). Several studies have also considered the effects of maximum temperature (Chan et al. 2011), minimum temperature (Ho et al. 2017), diurnal temperature range (Tam et al. 2009), and other thermal indices (Chau et al. 2022; Ho et al. 2022, 2024; Lee et al. 2016; Leung et al. 2008). A study on help-seeking behaviour of a senior citizen emergency hotline considered the effects of maximum, mean, and minimum temperatures, but only reported the effects of the stronger predictor, maximum temperature, on emergency health-related calls (Chan et al. 2011). However, there has not been a comprehensive and systematic analysis of the effects of different temperature indicators on adverse health outcomes in this high-density subtropical city.

This study conducted a comprehensive assessment on the associations of different temperature predictors (maximum, mean, and minimum temperatures) on mortality and hospital admission health outcomes during the 2010–2019 hot seasons in Hong Kong. The implications and significance of our study are applicable from both a local and global perspective. This assessment supports the update and decision-making of the heat-health warning system in Hong Kong using the latest heat-health impacts of the 2010–2019 hottest decade. On the other hand, this study adds to the global knowledge required to support decision-making for climate change adaptation and heat-health provision strategy development. The comprehensive assessment method in this study could be referred to by other cites to inform local decisions on heat warnings and subsequent interventions to mitigate the adverse effects of heat. Particularly, the application to other highly urbanized environments & subtropical cities would be useful to assess the generalizability of this study’s findings to other locations globally.

## Materials and methods

### Study area

Hong Kong is a coastal high-density city located in a sub-tropical climate zone. During the summer, Hong Kong experiences humid and hot conditions, with average afternoon temperatures exceeding 31 °C and night-time temperatures often remaining above 25 °C (Hong Kong Observatory 2021a). The city’s population is comprised of 15.9% older adults aged 65 and above (Census and Statistics Department 2017a), while over 19.9% of the population live under the poverty line (Census and Statistics Department 2017b). Air conditioning is used extensively in Hong Kong, taking up the largest category of residential electricity consumption at 36% in 2015 (Electrical & Mechanical Services Department, 2017). An earlier study in 2012 found that 90% of participants owned air conditioners (*N* = 1002), however, the usage was not equally distributed as those with low monthly household income were less likely to use air conditioning even when feeling hot (Gao et al. 2020).

### Data sources

Daily meteorological observations of maximum, mean, minimum temperatures, relative humidity, windspeed, total rainfall, and tropical cyclone days were obtained from the Hong Kong Observatory (HKO) for 2010–2019 for the hot season, which was defined as 15 May − 15 Oct. All measurements were taken from the HKO Headquarters located in the city centre. Daily data for air pollutants PM2.5, PM10, NO2, NOx, O3, SO2, and CO was collected from the Hong Kong Environmental Protection Department for the same period by a daily average across 13 general monitoring stations, apart from a rural monitoring station whose location was not reflective of the general population exposure.

Daily mortality and hospital admission data were obtained from the Census and Statistics Department and Hospital Authority for 2010–2019. The main analysis assessed non-cancer non-external causes, which included all principal diagnoses with International Classification of Diseases (ICD-9) code ranging from 001 to 139 and 240–799. Additionally, different disease groups were assessed in the stratification analysis, including circulatory (ICD-9 390–459), respiratory (ICD-9 460–519), and pneumonia and influenza (ICD-9 480–487). The outcomes were stratified by multiple age groups for comparison, including 15–64, 15–74, 65 and above, 65 to 74, 75 and above, all ages 15 and above. Additional age groups were calculated for mortality (75 to 84, 85 and above). Only non-scheduled hospital admissions occurring through the accident and emergency (A&E) department were included for analysis.

### Statistical analysis

We used maximum, mean, and minimum temperatures to examine their associations with non-cancer mortality and hospital admissions using the same time period and methodology across all models. A combination of quasi-Poisson Generalized Additive Models (GAM) and Distributed Lag Non-linear models (DLNM) was used to examine the associations, where DLNMs allowed for estimating the lag and cumulative effects of temperature. Non-linear temperature effect and lag structure with lag up to 12 days was examined. A crossbasis with natural spline was created for each temperature exposure based on the whole of May-Oct, with knots at 50th and 90th percentile of the hot seasons (15 May − 15 Oct). Optimal lag was determined for mortality and hospital admission outcomes separately, using a combination of quasi Akaike’s Information Criterion (qAIC) performance of different lag models and extended lag plot assessment to determine shortest ideal lag. The extended lag assessment was conducted with a lag of extended length (lag 0–12) to assess the lag day at which cumulative effect is reduced nearest to zero for multiple temperature slices. The optimal lag was set to lag 0–7 for mortality outcomes and lag 0–3 for hospital admissions.

The optimal temperature (OT) where the risk of mortality and hospitalization is at a minimum was pre-set using the median temperature for each temperature metric, then allowed to vary according to each model shape. This is also known as the minimum mortality temperature (MMT) or minimum hospitalization temperature (MHT) in other papers (Gasparrini et al. 2015; Lu et al. 2021; Sun et al. 2019; Tobias et al. 2021). For each model, we reported the temperatures associated with an increase in relative risk (RR) of 5% (RR:1.05) and 10% (RR:1.10), in line with the WHO-WMO 2015 Guidance for Warning-System Development and used by many warning systems as targets for issuing heat-health warnings (World Meteorological Organization (WMO) & World Health Organization (WHO) 2015). Relative risk is “the ratio of the risk of an event among the exposed to the risk among the unexposed” (*Oxford Handbook of Public Health Practice*^2013^). For comparison between the temperature indicators, we also reported the temperature percentile, which was calculated based on summer temperature distribution of the study period 2010–2019. Additionally, we reported the effect estimates of the 90th, 95th and 99th percentiles of the main temperature-health models in the Appendix (Tables A1, A2).

The analysis was controlled for rainfall and PM 2.5 (as a proxy for air pollutants), both square root transformed to reduce the impact of extreme values. The analysis was further adjusted for long-term trend (day of study, df = 3), seasonality (day of year, df = 3), day of week, and public holiday. Hospital admission models were additionally adjusted for days under effects of tropical cyclone when warnings are broadcasted for public safety and protection. Partial autocorrelation (PACF) plots and residual plots were used to assess the models and an autocorrelation of AR1 was added. The full formula is as follows:

$$\eqalign{& E\left( {\log \left( {{\rm{Daily}}\,\,{\rm{number}}\,\,{\rm{of}}\,\,{\rm{mortality}}\,\,{\rm{or}}\,\,{\rm{hospital}}} \right.} \right. \cr & \left. {\left. {\,\,\,\,\,\,\,\,\,\,\,\,\,\,\,\,\,\,{\rm{admissions}}\,\,{\rm{for}}\,\,{\rm{certain}}\,\,{\rm{age}}\,\,{\rm{group}}} \right)} \right) \cr & \,\,\,\,\,\,\,\,\,\,\,\,\,\,\,\,\, = {\rm{cb}}\left( {{\rm{temperature}}\,\,{\rm{exposure}},\,{\rm{lag}} = {\rm{optimal}}\,\,{\rm{lag}}} \right) \cr & \,\,\,\,\,\,\,\,\,\,\,\,\,\,\,\,\, + {\rm{s}}\left( {{\rm{sqrt}}{\rm{.}}\,\,{\rm{rain}},{\rm{k}} = 3} \right) + {\rm{s}}\left( {{\rm{sqrt}}{\rm{.}}\,\,{\rm{pm}}25,{\rm{k}} = 3} \right) \cr & \,\,\,\,\,\,\,\,\,\,\,\,\,\,\,\,\, + {\rm{s}}\left( {{\rm{dos}},{\rm{k}} = 3} \right) + {\rm{s}}\left( {{\rm{doy}},{\rm{k}} = 3} \right) + {\rm{factor}}\left( {{\rm{holiday}}} \right) \cr & \,\,\,\,\,\,\,\,\,\,\,\,\,\,\,\,\, + {\rm{factor}}\left( {{\rm{dow}}} \right) + {\rm{factor}}\left( {{\rm{tropical}}\,\,{\rm{cyclone}}} \right) \cr & \,\,\,\,\,\,\,\,\,\,\,\,\,\,\,\,\, + {\rm{autocorrelation}}\,\,{\rm{terms}} \cr} $$

where cb() indicates the crossbasis of temperature exposure using R package “dlnm”; s() indicates the smoothing function of continuous independent variables using R package “mgcv”; factor() indicates categorical independent variables; dos = day of study; doy = day of year; dow = day of week; tropical cyclone was adjusted in hospital admission models only; autocorrelation terms adjusted in this model: lag(outcome,1).

Stratification analyses were conducted to assess (1) the effects of sex on mortality outcomes only, and (2) the effects of disease-specific sub-groups (circulatory, respiratory, and pneumonia and influenza) on both mortality and hospital admissions. Due to data limitations, sex stratification was unavailable for hospital admissions. Sensitivity analyses were additionally conducted to assess the effects of longer lag (Lag 0–10), month, relative humidity, windspeed, and other pollutants (PM10, NO2, NOx, O3, SO2, and CO) on the model outcomes.

Statistical significance level was set at *p* ≤ 0.05, which was used to evaluate the evidence alongside the reporting of effect size, confidence interval, and exact p-values (Di Leo and Sardanelli 2020). All analyses were conducted with the statistical software R (version 3.5.2) (R Core Team 2018), using the dlnm() (Gasparrini 2011) and mgcv() (Wood 2017) packages.

Ethics approval was obtained from the Survey and Behavioural Ethics Committee of the Chinese University of Hong Kong.

## Results

The study period of 2010–2019 hot seasons from 15 May-15 Oct comprised of 1530 observation days. The ranges of daily maximum, mean, and minimum temperature during the study period was 22.1–36.6 °C, 20.5–32.4 °C, and 18.5–30.0 °C, respectively. The daily averages of non-cancer mortality, hospital admissions, and environmental variables can be seen on Table 1. Overall, the number of daily morbidity events was substantially higher than mortality events. Descriptive statistics for stratification and sensitivity analyses are found in Appendix, Tables A3 & A4.

**Table 1:** Descriptive statistics of mortality, hospital admission, and environmental variables in Hong Kong, 2010–2019 hot seasons (15 May − 15 Oct)

Variable	Min	Max	Mean (SD)
**Environmental variables**			
Maximum temperature (°C)	22.1	36.6	31.07 (2.10)
Mean temperature (°C)	20.5	32.4	28.39 (1.65)
Minimum temperature (°C)	18.5	30.0	26.45 (1.62)
Daily total rainfall (mm)	0	274	11.45 (26.42)
PM2.5 (µg/m^3^)	4.3	78	18.26 (12.78)
**Non-cancer mortality (daily counts)**			
15 to 64	0	18	8.02 (2.71)
15 to 74	4	33	16.30 (4.05)
65 and above	34	100	60.88 (9.31)
65 to 74	0	19	8.28 (2.88)
75 and above	27	86	52.61 (8.63)
75 to 84	7	39	20.70 (4.88)
85 and above	11	57	31.91 (7.35)
Overall (all ages 15+)	40	107	68.90 (9.80)
**Non-cancer hospital admissions (daily counts)**			
15 to 64	308	821	605.83 (71.79)
15 to 74	439	1124	821.78 (105.12)
65 and above	447	1117	819.48 (101.36)
65 to 74	120	351	215.95 (39.04)
75 and above	316	820	603.53 (67.65)
Overall (all ages 15+)	757	1899	1426.34 (165.40)

### Main analysis

**Fig. 1:**
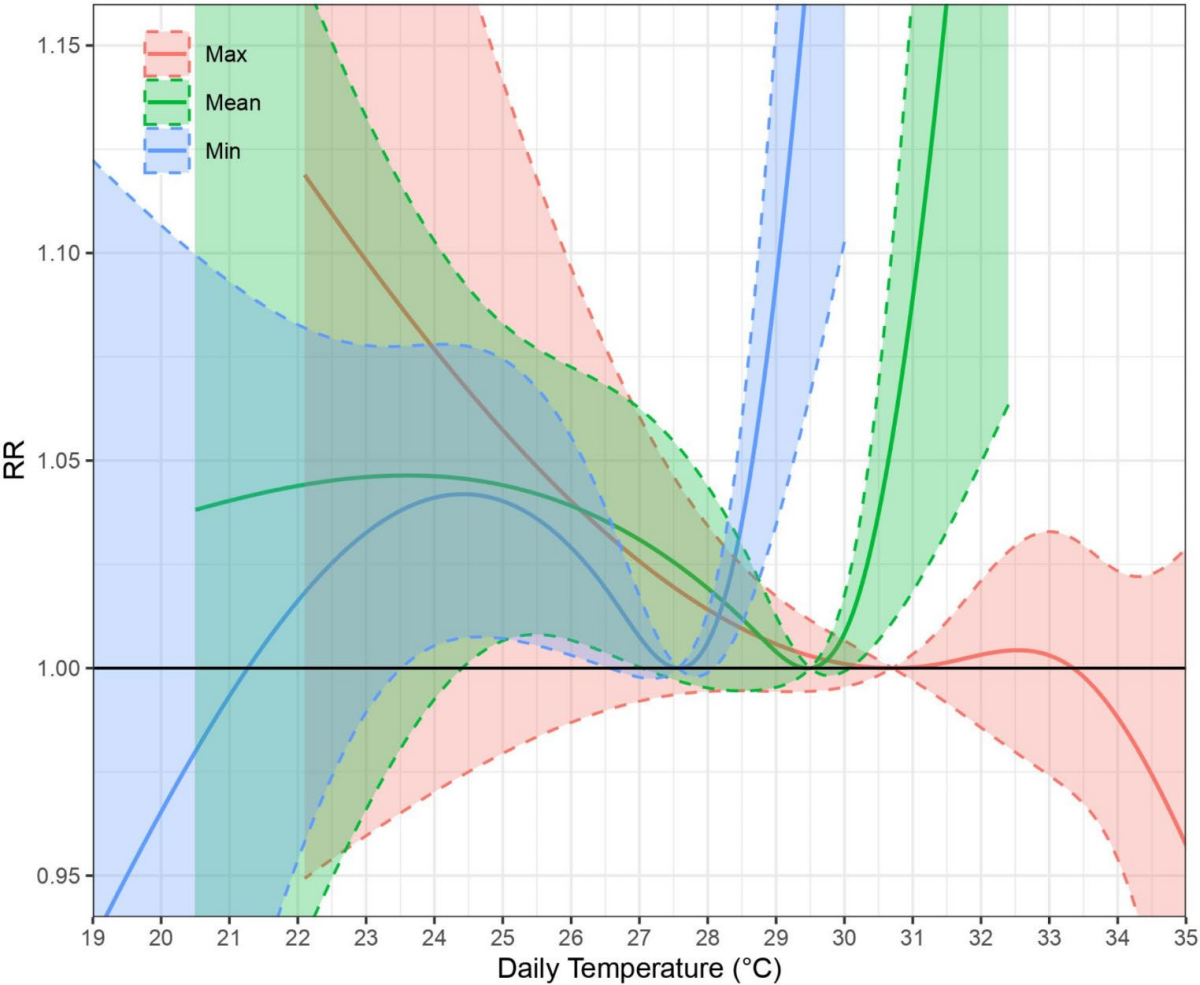
Cumulative associations of maximum, mean, and minimum temperatures on non-cancer mortality, 65 and above (Lag 0–7) RR = Relative Risk; Dotted lines = 95% confidence interval

Relative risk of non-cancer mortality increased with high temperatures for daily mean and minimum temperatures and among older adults 65 or above (see Table 2; Fig. 1). For daily mean temperatures of those 65 and above, the optimal temperature (OT) was 29.5 °C (69th percentile of the study period summer temperature distribution), with the 1.05 and 1.10 relative risk significant at 30.6 °C (95.9th percentile) and 31.1 °C (99.1st percentile) respectively. The relative risk (RR) for mean temperature at the 95th percentile (vs. OT) was 1.037 (95% Confidence Interval CI: 1.006, 1.069; p-value: 0.0189) (Appendix Table A1). For daily minimum temperatures of those 65 and above, the OT was 27.6 °C (71st percentile), with the 1.05 and 1.10 relative risk significantly associated at 28.6 °C (94.1st percentile) and 29.1 °C (97.6th percentile), respectively. The relative risk for minimum temperature at the 95th percentile (vs. OT) was 1.055 (95% CI: 1.019, 1.092; p-value: 0.0025) (Appendix Table A1). The variation between different older adult age cut-offs (75 and above or 85 and above, vs. 65 and above) led to a 0.1–0.3 °C difference of OT, 1.05, and 1.10 relative risk. Meanwhile, maximum temperatures did not find significant associations for mortality outcomes at any age group.

**Fig. 2:**
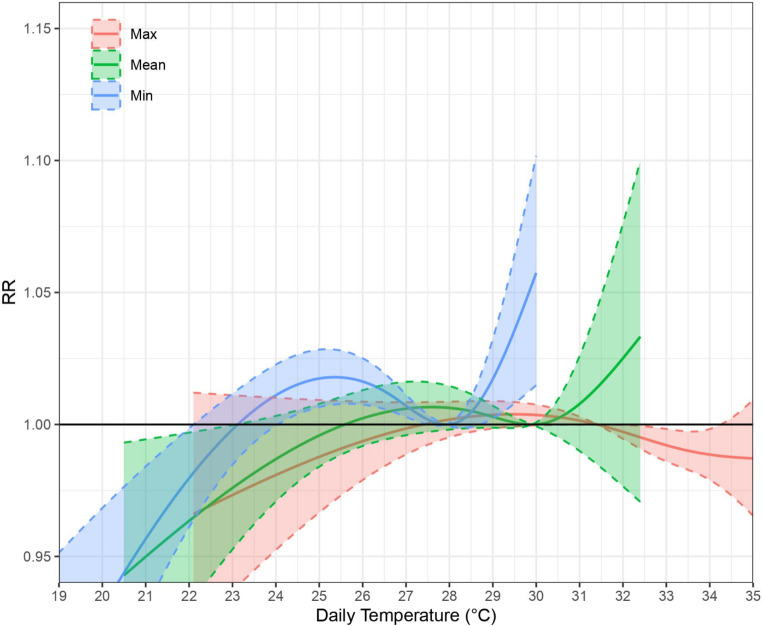
Cumulative associations of maximum, mean, and minimum temperatures on non-cancer hospital admissions, 65 and above (Lag 0–3) RR = Relative Risk; Dotted lines = 95% confidence interval

Relative risk of non-cancer hospital admissions increased with high temperatures for minimum temperatures and among older adult 65 or above (see Table 3; Fig. 2). For those 65 and above, OT of the minimum temperature model was 27.9 °C (80th percentile) with the 1.05 relative risk significantly associated at 29.8 °C (99.9th percentile). The relative risk for minimum temperature at the 95th percentile (vs. OT) was 1.009 (95% CI: 1.000, 1.018; p-value: 0.0486) (Appendix Table A2). A higher age cut-off of 75 and above led to a 0.1 °C difference of 1.05 relative risk. Maximum and mean temperatures were not significantly associated to the 1.05 or 1.10 relative risk with non-cancer hospitalizations outcomes of any age group.

**Table 2:** Mortality associations for each temperature metric, non-cancer (Lag 0–7)

	Maximum temperature			Mean temperature			Minimum temperature		
	OT (pct)	RR 1.05 (pct)	RR 1.10 (pct)	OT (pct)	RR 1.05 (pct)	RR 1.10 (pct)	OT (pct)	RR 1.05 (pct)	RR 1.10 (pct)
15 to 64	30.7 (39)	34.0 (95.6)	-	28.2 (40)	30.7 (96.9)	31.8 (99.9)	25.8 (34)	27.8 (76.6)	29.0 (97.6)
15 to 74	30.5 (35)	-	-	28.6 (48)	-	-	26.8 (55)	28.9 (96.9)	29.7 (99.9)
65 and above	30.7 (39)	-	-	29.5 (69)	30.6 (95.9)*	31.1 (99.1)*	27.6 (71)	28.6 (94.1)*	29.1 (97.6)*
65 to 74	30.2 (31)	-	-	28.7 (50)	-	-	27.8 (77)	29.1 (97.9)	29.6 (99.9)
75 and above	30.8 (40)	-	-	29.4 (68)	30.6 (95.7)*	31.0 (98.6)*	27.6 (71)	28.6 (94.1)*	29.0 (97.7)*
75 to 84	31.4 (50)	-	-	29.6 (74)	30.6 (96.6)	31.0 (98.6)	27.8 (76)	28.8 (95.4)	29.1 (98.2)
85 and above	30.2 (31)	-	-	29.3 (64)	30.5 (95.6)*	31.0 (98.6)*	27.4 (68)	28.5 (92.5)*	28.9 (96.8)*
Overall	30.9 (42)	-	-	29.4 (68)	30.7 (97.1)*	31.2 (99.3)*	27.5 (69)	28.7 (94.5)*	29.1 (98.1)*

OT = optimal temperature; RR = relative risk; pct = temperature percentile of summer temperature distribution; * = statistically significant, *p* ≤ 0.05 (exact p-values reported in Appendix Table A1). Models adjusted for long-term trend (day of study), seasonality (day of year), day of week, holiday, rainfall, PM2.5, and autocorrelation of AR 1

**Table 3:** Hospital admissions associations for each temperature metric, non-cancer (Lag 0–3)

	Maximum temperature			Mean temperature			Minimum temperature		
	OT (pct)	RR 1.05 (pct)	RR 1.10 (pct)	OT (pct)	RR 1.05 (pct)	RR 1.10 (pct)	OT (pct)	RR 1.05 (pct)	RR 1.10 (pct)
15 to 64	31.4 (50)	-	-	28.7 (50)	-	-	26.6 (50)	-	-
15 to 74	31.4 (50)	-	-	28.7 (50)	-	-	27.7 (75)	-	-
65 and above	31.4 (50)	-	-	29.9 (81)	-	-	27.9 (80)	29.8 (99.9)*	-
65 to 74	31.4 (50)	-	-	30.4 (93)	-	-	28.1 (84)	-	-
75 and above	31.4 (50)	-	-	29.8 (77)	-	-	27.9 (79)	29.7 (99.9)*	-
Overall	31.4 (50)	-	-	29.7 (75)	-	-	27.8 (78)	-	-

OT = optimal temperature; RR = relative risk; pct = temperature percentile of summer temperature distribution; * = statistically significant, *p* ≤ 0.05 (exact p-values reported in Appendix Table A2). Models adjusted for long-term trend (day of study), seasonality (day of year), day of week, holiday, rainfall, PM2.5, tropical cyclone, and autocorrelation of AR 1

### Stratification and sensitivity analyses

When non-cancer mortality outcomes were stratified by sex, the associations remained significant among the female sex only (Appendix Table A5). For mean temperatures, a 9-percentile difference between the sexes was found for the OT, although the difference diminished with the RR thresholds. This difference in OT and relative risk thresholds between females and males decreased with older age cut-offs. For minimum temperature, up to 6-percentile difference between the sexes was found for the RR thresholds and the difference between females and males remained similar with older age cut-offs.

In disease-specific sub-group mortality analyses (Appendix Table A6), significant associations were found for pneumonia and influenza mortality in maximum (for those 85 and above only), mean, and minimum temperatures, and for respiratory mortality in minimum temperatures only. In disease-specific hospital admissions (Appendix Table A7), significantly associations were found for circulatory disease admissions (75 and above) and pneumonia and influenza admissions (all ages).

The sensitivity analyses found similar outcomes for mortality (Appendix Table A8) and hospital admissions (Appendix Table A9) after adjusting for longer lags, relative humidity, windspeed, month (instead of day of year), and other pollutants besides PM2.5 (such as PM10, NO2, NOx, O3, SO2, and CO). The qAIC and Adjusted R^2^ did not demonstrate substantial improvements for the sensitivity models.

## Discussion

Our study found that mean and minimum temperatures were associated with increased risk of mortality, while minimum temperatures were associated with increased risk of hospital admissions in Hong Kong. Maximum temperatures were not found significantly associated with either mortality or hospital admissions. For mortality outcomes of both mean and minimum temperatures, the OT was located around the 70th percentile (of the summer temperature distribution), while the 1.05 and 1.10 relative risk was around 94-96th percentile and 97-99th percentile, respectively. For hospital admissions, OT was at the 80th percentile of minimum temperatures, with the 1.05 relative risk at 99.9th percentile. The effect was mainly seen in older adults 65 and above, with higher age cut-offs of 75 and above or 85 and above demonstrating a slight decrease of OT and risk thresholds. When stratified by other disease groups and sex, the effect was mainly seen among female sex and respiratory or pneumonia and influenza outcomes.

### Comparison with other comparative studies

Mean and minimum temperatures were found to be strong indicators for adverse health outcomes of mortality and hospital admissions in this high-density sub-tropical city of Hong Kong. Although other comparative studies previously concluded no temperature predictor is superior (Barnett et al. 2010; Kim et al. 2011; Schaeffer et al. 2016), our findings were similar to other cities where increased mortality risk was more strongly associated with mean temperatures in Brisbane, Australia (Vaneckova et al. 2011; Yu et al. 2011); Sao Paulo, Brazil (Gouveia et al. 2003); Tianjin China (Guo et al. 2011); and a multi-city study on London (UK), Budapest (Hungary), and Milan (Italy) (Hajat et al. 2006). Several studies also found strong effects of mortality with minimum temperatures in Houston, Texas USA (Heaton et al. 2014), Melbourne (Nicholls et al. 2008), and Brisbane, Australia (Yu et al. 2011). Yu et al. (2011) found that mean temperature was better indicator for all-age mortality, while adults 0–64 and older adults 65–74 and 85 + were more sensitive to minimum temperature in subtropical Brisbane, Australia. Our study found that mortality among older adults were sensitive to both mean and minimum temperatures, which drove most of the effect in the all-ages mortality.

Studies globally have found that mortality risk was more strongly associated with temperatures than morbidity or hospitalization risk (Astrom et al. 2011; Bunker et al. 2016; Dang et al. 2019; Iniguez et al. 2021; Kovats et al. 2004). Our study likewise found stronger impact of temperatures on mortality compared to hospital admissions. As heat can have an immediate effect on health, a rapid progression towards mortality may occur before individuals have a chance to obtain medical intervention (Bunker et al. 2016; Iniguez et al. 2021; Kovats et al. 2004). As such, public health efforts should be directed at strengthening the support and protection at the community level (Kovats et al. 2004), with mortality serving as an important endpoint of how well heat protective measures are working in the population.

### Effects of sex, disease sub-groups, and relative humidity

The female sex was more strongly associated with adverse health outcomes in extreme heat, which is in agreement with majority of evidence (Astrom et al. 2011; Basu 2009; Chan et al. 2013; van Steen et al. 2019). However, exact physiological or socio-cultural factors for the sex difference remains to be examined (van Steen et al. 2019). Among disease-specific subgroups, our study found significant associations mostly for respiratory and pneumonia and influenza outcomes. Interestingly, we found cardiovascular-related mortality associated with mean temperatures (albeit non-significantly), but cardiovascular-related admissions associated with minimum temperatures for those 75 and above. In previous systematic reviews, high temperatures were associated with increased risk for cardiovascular mortality but not cardiovascular morbidity without specifying the temperature indicator used (Bunker et al. 2016; Song et al. 2017). Our study findings suggest potentially different factors influencing cardiovascular-related mortality and hospital admission outcomes and further research is needed to understand the epidemiology and underlying mechanisms of these cardiovascular-related outcomes.

Relative humidity did not have a significant effect in the sensitivity analysis, consistent with findings from several other comparison studies (Barnett et al. 2010; Schaeffer et al. 2016; Vaneckova et al. 2011). Some studies have advocated for simple temperature measures as a “sufficient measure of heat stress” (Nicholls et al. 2008) for easier understanding and communication with the general public (Barnett and Astrom 2012). In this paper, we were unable to compare more complex thermal indices that integrate temperature, humidity and other parameters into a single measure, such as Heat Index, Wet-Bulb Globe Temperature, or Universal Thermal Climate Index. Given the trend of a changing climate, densely populated cities in subtropical areas are getting hotter and wetter in the summers (He et al. 2023; McAllister et al. 2022; Xu et al. 2020) and the relationship of humidity in heat-health outcomes should be revisited.

### Implications for practice and research

#### Enhancement of hot weather warning systems

Our study findings suggest the potential benefits of implementing an extreme hot warning system and heat-health action plan. The lack of significant association between daily maximum temperature and the health outcomes in our analysis may be influenced by several factors, including the implementation of the Very Hot Weather Warning (VHWW) since 2000. While our study did not directly assess the temporal impact of the VHWW, its introduction by the Hong Kong Observatory (HKO) aimed to increase public awareness and promote preventive actions during very hot conditions with maximum temperatures of 33 °C or above. The heat stress information service for Hong Kong was further enhanced in 2014, when HKO began to issue Hot Weather Special Advisory in marginally very hot conditions, where the VHWW threshold is not yet attained but may still bring elevated health risk (Lee et al. 2016). A previous study estimated that the absence of VHWW on hot days during its early years of implementation was associated with an increased risk of 1.63 and 1.23 for daily IHD and stroke mortality, respectively (Chau et al. 2009). Our study findings are supportive of this assessment, suggesting that the VHWW could effectively reduce the heat health risk under very hot weather in Hong Kong. Furthermore, the HKO enhanced the VHWW, which is disseminated via multiple channels, in 2022 with a prolonged heat alert when a few consecutive hot nights (minimum temperature at least 28 °C) are expected (Hong Kong Observatory 2022).

>Further potential use and implementation of mean or minimum temperatures in the enhancement of heat-health warning systems or real-time heat-health surveillance systems can be explored, as several countries have also included minimum temperatures as part of the indicators to trigger their heat-health warning systems, such as Austria, Belgium, England, France, Germany, Spain, and India (Casanueva et al. 2019; Kotharkar and Ghosh 2022). The setting of temperature indicators (including the maximum temperature) and trigger thresholds within the heat-health warning systems should be monitored and periodically reviewed to understand its effectiveness and relevance under the warming conditions brought about by climate change, urbanization, and changing demographics.

#### Climate change adaptation strategies

Minimum temperatures during the hot season typically coincide with the lowest night-time temperatures. Our study’s findings regarding minimum temperatures are consistent with existing literature that links high night-time temperatures to increased mortality risk (He et al. 2022; Laaidi et al. 2012; Murage et al. 2017), particularly among the elderly and in urban areas (Laaidi et al. 2012). Additionally, we found that minimum temperatures were more sensitive than mean temperatures in capturing the effects on hospital admissions. This aligns with Chen et al. (2017), which found that minimum temperatures demonstrated stronger and more frequent associations for emergency department visits in Atlanta, Georgia, USA. Minimum temperatures may reflect conditions “*where little relief is available to persons under elevated night-time temperatures*” (Kinney et al. 2008). High night-time temperatures may affect the body’s circadian thermoregulation, interrupt the regular sleep physiology, and result in poorer sleep quality (Obradovich et al. 2017; Okamoto-Mizuno et al. 1999). The lack of relief may also affect people’s capacity to handle extreme heat the next day (Perkins 2015). Particularly, highly dense urban areas may experience an amplification of night-time temperatures due to the urban heat island effect, where stored daytime heat in the urban infrastructure is released overnight (Arnfield 2003). The urban heat island intensity is greatest at night and known to be intensified with higher population density and larger city size (Arnfield 2003). As a highly dense city, Hong Kong is populated by up to 57,530 per square kilometre in some districts (Census and Statistics Department 2017a). Previous studies by our team have found that extreme heat events affected the spatial variation and intensity of the local urban heat island effect, leading to increased mortality risk (Ho et al. 2023; Ren et al. 2021). With climate change, night-time temperatures are projected to increase in cities around this region (He et al. 2022), particularly coastal areas. Hence, night-time heat will be an important extreme weather impact to adapt and address in the coming years given the future changing climate, especially for other similar subtropical cities and highly urbanized areas. Different adaptation strategies may include: (1) improving urban ventilation and designing buildings and neighbourhoods to mitigate night-time UHI effect and relieve heat stress in densely populated settings like Hong Kong; (2) creating and opening heat-sheltering facilities at the community level especially in areas of high heat risk; and (3) increasing awareness and public education of heat-health and heat-resilience issues at the societal and community level. For example, the Hong Kong Observatory has published educational videos through the Cool Met Stuff programme since 2014.

#### Heat-related illness prevention and action plans

As night-time warming is increasing rapidly in many parts of the world (Cox et al. 2020), this study has important implications for cities globally to not only address the heat risk during day-time but also night-time. Moving forward, heat-related illness prevention and action plans need make provisions and long-term preparations to tackle the difficulties of addressing night-time heat. Increased awareness of night-time heat and heat-related sleep quality may be promoted in the population. Recommendations for heat prevention need to consider and include night-time strategies, including (1) maintaining ventilation and air circulation at night, (2) adjusting sleepwear and bedding, (3) turning on AC/fan during the night or for several hours before bedtime, (4) moving to a cooler area of the room or home, (5) staying hydrated during the day, and (6) knowing signs of heat-related illnesses, and (7) monitoring high-risk populations including those 65 and above. More recommendations have been developed by the European Insomnia Network (Altena et al. 2023). Apart from actions from government side, relevant protocols should be developed for organizations and NGOs, particularly those involved with elderly care.

#### Future research directions

Our findings echo the recent global studies assessing the impacts of night-time warming on health (He et al. 2022). Future studies can explore the use, implementation, and effectiveness of mean or minimum temperature as an indicator within health-health warning systems, particularly in sub-tropical high-density settings. Future research can further assess whether the implementation of the Very Hot Weather Warning in the year 2000 had an effect to alter the health associations of maximum temperatures post-implementation. More studies could examine the mechanisms of night-time temperatures related to the increased risk of mortality and hospital admissions. Mechanisms for differences in sex and disease-specific sub-groups could additionally be assessed.

### Strengths and limitations

To the author’s knowledge, this was the first study to comprehensively evaluate multiple temperature indicators on adverse health outcomes of mortality and hospital admissions in a subtropical city during this hottest decade on record. Our study included multiple age cut-offs and stratification by sex and different disease groups to provide a comprehensive understanding of hot temperature associations. We used a consistent robust statistical analysis for comparison across all temperature models and adjusted for other meteorological and air pollutant variables.

However, this study has its share of limitations. Our study was located in a single highly urbanized setting and the findings may not be generalizable to other settings, particularly locations with different climates, demographics, heat adaptations, healthcare systems, and urbanization (Zhang et al. 2023). We did not examine the potential modifiers of association between temperature and health outcomes, including but not limited to the use of air conditioning and other heat mitigation measures. This study also did not account for the effect of the duration of extreme hot weather (Song et al. 2023; Wang et al. 2019), compound extreme events (He et al. 2023; Huang et al. 2023), and within-city spatial variations due to varying levels of vulnerability (Ho et al. 2024), urbanization (Xing et al. 2020, 2022), or the urban heat island effect (Ho et al. 2023; Xin et al. 2023). Furthermore, as an observational study, this study cannot determine causation of the findings (e.g., the effectiveness of VHWW on reducing heat health risk). The findings may be subject to ecological fallacy. Finally, the temperature and other meteorological data from the singular station at the city centre may or may not reflect individual exposures.

## Conclusions

Using a comprehensive assessment method, mean and minimum temperatures were identified as strong indicators for adverse health outcomes in hot temperatures in the subtropical city of Hong Kong. Mean temperatures were associated with mortality, while minimum temperatures were associated with both mortality and hospital admissions. Maximum temperatures did not demonstrate a significant effect for either mortality or hospital admissions. Our study recommends the use of a comprehensive assessment method to evaluate the local heat-health risk and support the strengthening of heat-health warning systems and heat prevention strategies. With the projected increase of night-time warming under climate change, actions to mitigate the health risks associated with rising night temperatures will be essential. Our paper discussed the implications of these study findings on hot weather warning systems, climate change adaptation strategies, and heat-related illness prevention and action plans. Utilizing mean or minimum temperatures as key indicators will be vital in shaping effective adaptation strategies and improving the implementation of heat-health warning systems in the future.

## Electronic supplementary material

Below is the link to the electronic supplementary material.

Supplementary Material 1

## Data Availability

The data that support the findings of this study are available from Census and Statistics Department and Hospital Authority, Hong Kong but restrictions apply to the availability of these data, which were used under license for the current study and so are not publicly available. The data are, however, available from the authors upon reasonable request and with the permission of Census and Statistics Department and Hospital Authority, Hong Kong.
