# Supplementary material for: Suitable temperature indicator for adverse health impacts in sub-tropical cities: a case study in Hong Kong from 2010-2019
Source: Int J Biometeorol. 2024 Oct 30;69(1):233–44. doi: 10.1007/s00484-024-02807-1 (PMC11680666; doi:10.1007/s00484-024-02807-1)
Supplement: Supplementary file 1 — Supplementary Material 1 [file 484_2024_2807_MOESM1_ESM.docx]

**Title:** **Suitable temperature indicator for adverse health impacts in sub-tropical cities: a case study in Hong Kong from 2010-2019**

**Authors:** Janice Ying-en Ho, Yitong Guo, Ka Chun Chong, Pak Wai Chan, Chun Kit Ho, Hiu Fai Law, Ren Chao, Edward Yan Yung Ng, Kevin Ka Lun Lau

**Appendix**

|  | Page |
| --- | --- |
| Table A.1. Effect estimates of main temperature-mortality associations | 2 |
| Table A.2. Effect estimates of main temperature-hospitalization associations | 3 |
| Table A.3. Descriptive statistics of sex-stratified and disease-specific mortality and hospital admissions in Hong Kong, 2010-2019 hot seasons | 4 |
| Table A.4. Descriptive statistics of environmental variables in sensitivity analysis | 5 |
| Table A.5. Sex-stratified non-cancer mortality associations for maximum, mean, and minimum temperatures | 6 |
| Table A.6. Disease-specific mortality associations for maximum, mean, and minimum temperatures | 7 |
| Table A.7. Disease-specific hospital admission associations for maximum, mean, and minimum temperatures | 8 |
| Table A.8. Sensitivity analyses of non-cancer mortality associations for each temperature metric | 9 |
| Table A.9. Sensitivity analyses of non-cancer hospital admission associations for each temperature metric | 11 |

**Table A.1. Effect estimates of main temperature-mortality associations**

| **Non-cancer mortality** | **Temperature** | **OT** | **90^th^ percentile** | **p-value** | **95^th^ percentile** | **p-value** | **99^th^ percentile** | **p-value** |
| --- | --- | --- | --- | --- | --- | --- | --- | --- |
| 65 and above | Maximum temp | 30.70 (37.8) | 0.999 (0.969, 1.031) | 0.9541 | 0.990 (0.958, 1.024) | 0.5658 | 0.959 (0.894, 1.028) | 0.2425 |
|  | Mean temp | 29.44 (68.9) | 1.017 (1.001, 1.033)* | 0.0354 | 1.037 (1.006, 1.069)* | 0.0189 | 1.097 (1.020, 1.180)* | 0.0127 |
|  | Min temp | 27.57 (71.0) | 1.027 (1.007, 1.047)* | 0.0074 | 1.055 (1.019, 1.092)* | 0.0025 | 1.140 (1.053, 1.235)* | 0.0013 |
| 75 and above | Maximum temp | 30.80 (39.9) | 1.003 (0.971, 1.036) | 0.8662 | 0.998 (0.964, 1.034) | 0.9181 | 0.981 (0.910, 1.057) | 0.6282 |
|  | Mean temp | 29.44 (68.9) | 1.021 (1.004, 1.038)* | 0.0144 | 1.045 (1.011, 1.079)* | 0.0080 | 1.114 (1.031, 1.204)* | 0.0064 |
|  | Min temp | 27.57 (71.0) | 1.030 (1.009, 1.051)* | 0.0045 | 1.060 (1.022, 1.100)* | 0.0020 | 1.153 (1.059, 1.255)* | 0.0011 |
| Overall | Maximum temp | 30.90 (41.5) | 1.001 (0.975, 1.028) | 0.9462 | 0.994 (0.965, 1.024) | 0.7041 | 0.967 (0.906, 1.033) | 0.3207 |
|  | Mean temp | 29.44 (68.9) | 1.016 (1.001, 1.031)* | 0.0348 | 1.034 (1.005, 1.063)* | 0.0194 | 1.085 (1.014, 1.161)* | 0.0180 |
|  | Min temp | 27.49 (69.0) | 1.027 (1.008, 1.046)* | 0.0048 | 1.052 (1.018, 1.087)* | 0.0025 | 1.128 (1.046, 1.215)* | 0.0017 |

OT = optimal temperature; * = statistically significant, p≤0.05. Models adjusted for long-term trend (day of study), seasonality (day of year), day of week, holiday, rainfall, and PM2.5.

**Table A.2. Effect estimates of main temperature-hospitalization associations**

| **Non-cancer hospitalizations** | **Temperature** | **OT** | **90^th^ percentile** | **p-value** | **95^th^ percentile** | **p-value** | **99^th^ percentile** | **p-value** |
| --- | --- | --- | --- | --- | --- | --- | --- | --- |
| 65 and above | Maximum temp | 31.40 (50) | 0.990 (0.983, 0.997)* | 0.0054 | 0.989 (0.980, 0.998)* | 0.0171 | 0.987 (0.966, 1.009) | 0.2413 |
|  | Mean temp | 29.90 (80.9) | 1.001 (0.998, 1.003) | 0.4414 | 1.002 (0.995, 1.010) | 0.6131 | 1.009 (0.989, 1.028) | 0.3700 |
|  | Min temp | 27.90 (78.3) | 1.003 (0.999, 1.007) | 0.1413 | 1.009 (1.000, 1.018)* | 0.0486 | 1.028 (1.005, 1.051)* | 0.0155 |
| 75 and above | Maximum temp | 31.40 (50) | 0.992 (0.984, 1.000) | 0.0505 | 0.990 (0.981, 1.000)* | 0.0397 | 0.988 (0.965, 1.012) | 0.3245 |
|  | Mean temp | 29.80 (79.8) | 1.001 (0.998, 1.005) | 0.5871 | 1.004 (0.996, 1.012) | 0.3312 | 1.013 (0.991, 1.035) | 0.2464 |
|  | Min temp | 27.90 (80.6) | 1.004 (0.999, 1.009) | 0.1161 | 1.011 (1.001, 1.020)* | 0.0224 | 1.032 (1.008, 1.057)* | 0.0093 |
| Overall | Maximum temp | 31.40 (50) | 0.994 (0.988, 1.000) | 0.0503 | 0.992 (0.985, 1.000)* | 0.0369 | 0.988 (0.970, 1.006) | 0.1954 |
|  | Mean temp | 29.70 (75.9) | 1.001 (0.998, 1.004) | 0.5239 | 1.002 (0.995, 1.009) | 0.5871 | 1.007 (0.990, 1.024) | 0.4260 |
|  | Min temp | 27.80 (75.6) | 1.003 (0.999, 1.007) | 0.1413 | 1.007 (1.000, 1.015) | 0.0659 | 1.022 (1.003, 1.041)* | 0.0000 |

OT = optimal temperature; * = statistically significant, p≤0.05. Models adjusted for long-term trend (day of study), seasonality (day of year), day of week, holiday, rainfall, PM2.5, and tropical cyclone.

**Table A.3. Descriptive statistics of sex-stratified and disease-specific mortality and hospital admissions in Hong Kong, 2010-2019 hot seasons**

| **Sex stratification** | | **Female** | | | **Male** | | |
| --- | --- | --- | --- | --- | --- | --- | --- |
|  | **Age groups** | **Min** | **Max** | **Mean (SD)** | **Min** | **Max** | **Mean (SD)** |
| Non-cancer mortality | 65 and above | 13 | 50 | 30.33 (6.17) | 14 | 59 | 30.55 (6.15) |
|  | 75 and above | 10 | 48 | 27.85 (5.87) | 10 | 54 | 24.75 (5.58) |
|  | 85 and above | 4 | 35 | 19.46 (5.15) | 2 | 28 | 12.45 (4.09) |
|  | Overall | 17 | 54 | 32.61 (6.36) | 16 | 69 | 36.29 (6.69) |
|  |  |  |  |  |  |  |  |
| **Other Disease groups** | | **Mortality** | | | **Hospital admissions** | | |
| **Diagnoses groups** | **Age groups** | **Min** | **Max** | **Mean (SD)** | **Min** | **Max** | **Mean (SD)** |
| Circulatory | 15 to 64 | 0 | 11 | 3.81 (1.92) | 24 | 100 | 58.06 (10.36) |
|  | 15 to 74 | 1 | 18 | 7.07 (2.60) | 53 | 153 | 94.71 (15.39) |
|  | 65 and above | 7 | 38 | 19.46 (4.72) | 76 | 191 | 134.14 (17.70) |
|  | 65 to 74 | 0 | 11 | 3.26 (1.76) | 14 | 71 | 36.64 (8.08) |
|  | 75 and above | 3 | 33 | 16.21 (4.32) | 47 | 138 | 97.50 (12.99) |
|  | 75 to 84 | 0 | 20 | 7.17 (2.88) | / | / | / |
|  | 85 and above | 0 | 21 | 9.04 (3.24) | / | / | / |
|  | Overall | 9 | 43 | 23.27 (5.16) | 100 | 284 | 192.46 (24.14) |
| Respiratory | 15 to 64 | 0 | 6 | 1.44 (1.16) | 21 | 118 | 50.63 (12.25) |
|  | 15 to 74 | 0 | 12 | 3.91 (1.95) | 37 | 187 | 84.38 (18.70) |
|  | 65 and above | 7 | 44 | 22.61 (5.53) | 89 | 338 | 165.09 (32.88) |
|  | 65 to 74 | 0 | 10 | 2.46 (1.57) | 16 | 81 | 33.75 (8.73) |
|  | 75 and above | 7 | 41 | 20.15 (5.20) | 73 | 268 | 131.35 (26.70) |
|  | 75 to 84 | 0 | 20 | 7.36 (2.81) | / | / | / |
|  | 85 and above | 2 | 30 | 12.78 (4.14) | / | / | / |
|  | Overall | 9 | 45 | 24.05 (5.71) | 110 | 455 | 215.89 (42.22) |
| Pneumonia and Influenza | 15 to 64 | 0 | 6 | 0.99 (0.97) | 2 | 81 | 19.50 (8.12) |
|  | 15 to 74 | 0 | 10 | 2.62 (1.60) | 12 | 122 | 32.56 (12.23) |
|  | 65 and above | 4 | 37 | 17.43 (5.17) | 45 | 229 | 84.67 (23.54) |
|  | 65 to 74 | 0 | 7 | 1.63 (1.29) | 3 | 49 | 13.06 (5.50) |
|  | 75 and above | 4 | 35 | 15.79 (4.87) | 37 | 188 | 71.61 (19.63) |
|  | 75 to 84 | 0 | 15 | 5.26 (2.35) | / | / | / |
|  | 85 and above | 1 | 27 | 10.53 (3.90) | / | / | / |
|  | Overall | 5 | 39 | 18.42 (5.34) | 54 | 310 | 104.26 (29.82) |

**Table A.4. Descriptive statistics of environmental variables in sensitivity analysis**

| **Variable** | **Min** | **Max** | **Mean (SD)** |
| --- | --- | --- | --- |
| **Environmental variables** |  |  |  |
| Relative Humidity (%) | 40 | 98 | 80.41 (7.68) |
| Wind (km/h) | 4.4 | 102 | 21.00 (10.21) |
| PM10 (µg/m^3^) | 7.6 | 108 | 28.50 (16.75) |
| NO2 (µg/m^3^) | 4.1 | 124 | 42.93 (15.90) |
| NOx (µg/m^3^) | 6.4 | 212 | 75.79 (28.45) |
| O3 (µg/m^3^) | 6.5 | 165 | 38.88 (26.24) |
| SO2 (µg/m^3^) | 3.3 | 45 | 9.73 (5.22) |
| CO (µg/m^3^) | 18.7 | 114 | 56.35 (15.00) |

**Table A.5. Sex-stratified non-cancer mortality associations for maximum, mean, and minimum temperatures**

|  | **Maximum temp** | | | **Mean temp** | | | **Minimum temp** | | |
| --- | --- | --- | --- | --- | --- | --- | --- | --- | --- |
|  | **OT (pct)** | **RR 1.05 (pct)** | **RR 1.10 (pct)** | **OT (pct)** | **RR 1.05 (pct)** | **RR 1.10 (pct)** | **OT (pct)** | **RR 1.05 (pct)** | **RR 1.10 (pct)** |
| **65 and above, female** | 30.3 (33) | - | - | 29.4 (66) | 30.4 (93.1)* | 30.8 (97.7)* | 27.6 (71) | 28.5 (91.2)* | 28.8 (95.5)* |
| **65 and above, male** | 31.4 (50) | - | - | 29.7 (75) | 31.0 (98.9) | 31.6 (99.8) | 27.6 (73) | 29.0 (97.6) | 29.6 (99.7) |
| **75 and above, female** | 30.3 (33) | - | - | 29.3 (65) | 30.4 (93.1)* | 30.8 (97.5)* | 27.5 (70) | 28.4 (91.2)* | 28.7 (95.4)* |
| **75 and above, male** | 33.5 (92) | - | - | 29.6 (74) | 30.7 (97.2) | 31.1 (99.2) | 27.7 (74) | 29.0 (97.7) | 29.5 (99.6) |
| **85 and above, female** | 30.3 (33) | - | - | 29.3 (64) | 30.5 (95.1)* | 30.9 (98.1)* | 27.5 (70) | 28.4 (90.3)* | 28.7 (95.4)* |
| **85 and above, male** | 31.4 (50) | - | - | 29.3 (64) | 30.6 (96.1) | 31.1 (99.0) | 26.6 (50) | 28.8 (96.4) | 29.5 (99.7) |
| **Overall, female** | 30.3 (33) | - | - | 29.3 (65) | 30.5 (93.1)* | 30.9 (98.1)* | 27.5 (70) | 28.5 (91.2)* | 28.8 (96.2)* |
| **Overall, male** | 31.4 (50) | - | - | 29.6 (74) | 31.1 (99.2) | 31.8 (99.9) | 27.5 (70) | 29.1 (98.2) | 29.8 (99.9) |

For mean temperature models, females 65 and above reported an OT of 29.4°C (66th percentile) in the mean temperature model, with statistically significant 5% and 10% relative risk at 30.4°C and 30.8°C, respectively. Meanwhile, males 65 and above reported a higher OT of 29.7°C (75th percentile) and a higher but non-significant 5% and 10% relative risk at 31.0°C and 31.6°C, respectively. A 9-percentile difference between sexes was found for the OT, although the difference diminished with the RR thresholds. This difference in OT and relative risk thresholds between females and males seemed to decrease with older age cut-off.

For minimum temperature models, females 65 and above reported an OT of 27.6°C, with statistically significant 5% and 10% relative risk at 28.5°C and 28.8°C, respectively. Males 65 and above reported 27.6°C OT, with higher but non-significant 5% and 10% relative risk at 29.0°C and 29.6°C, respectively. Up to 6-percentile difference between sexes was found for the RR thresholds and the difference between females and males remained similar with older age cut-offs.

**Table A.6. Disease-specific mortality associations for maximum, mean, and minimum temperatures**

| **Diagnoses groups** | **Age groups** | **Maximum temp** | | | **Mean temp** | | | **Minimum temp** | | |
| --- | --- | --- | --- | --- | --- | --- | --- | --- | --- | --- |
|  |  | OT (pct) | RR 1.05 (pct) | RR 1.10 (pct) | OT (pct) | RR 1.05 (pct) | RR 1.10 (pct) | OT (pct) | RR 1.05 (pct) | RR 1.10 (pct) |
| Circulatory | 15 to 64 | 31.4 (50) | - | - | 28.7 (50) | - | - | 26.6 (50) | 27.3 (66.8)* | - |
|  | 15 to 74 | 31.4 (50) | - | - | 27.4 (26) | - | - | 26.6 (50) | - | - |
|  | 65 and above | 31.4 (50) | - | - | 29.5 (71) | 30.6 (95.9) | 31.1 (99.0) | 28.6 (93) | - | - |
|  | 65 to 74 | 30.3 (33) | - | - | 28.7 (50) | - | - | 26.6 (50) | - | - |
|  | 75 and above | 32.8 (80) | 35.5 (99.8) | - | 29.5 (71) | 30.5 (95.6) | 30.9 (98.2) | 28.0 (81) | - | - |
|  | 75 to 84 | 33.0 (84) | 34.8 (98.8) | 35.6 (99.8) | 29.6 (73) | 30.5 (94.6) | 30.8 (98.1) | 26.6 (50) | - | - |
|  | 85 and above | 32.2 (65) | - | - | 29.4 (67) | 30.6 (95.7) | 31.0 (98.6) | 26.6 (50) | 29.6 (99.7) | - |
|  | Overall | 31.4 (50) | - | - | 29.5 (69) | 31.2 (99.3) | 31.9 (99.9) | 26.0 (38) | - | - |
| Respiratory | 15 to 64 | 31.1 (44) | 35.9 (99.9) | - | 29.7 (75) | 30.4 (94.1) | 30.6 (95.9) | 27.8 (78) | 28.7 (94.5) | 29.0 (97.6) |
|  | 15 to 74 | 31.3 (48) | - | - | 30.0 (83) | 31.1 (99.2) | 31.6 (99.8) | 27.7 (75) | 28.7 (95.4) | 29.1 (98.2) |
|  | 65 and above | 30.4 (34) | - | - | 29.5 (69) | 30.6 (95.9) | 31.1 (99.0) | 27.6 (71) | 28.4 (90.3)* | 28.7 (95.4)* |
|  | 65 to 74 | 31.4 (50) | - | - | 28.7 (50) | - | - | 27.6 (72) | 28.7 (94.5) | 29.1 (97.9) |
|  | 75 and above | 30.4 (34) | - | - | 29.5 (69) | 30.5 (95.6) | 30.9 (98.5) | 27.6 (71) | 28.4 (90.9)* | 28.7 (94.5)* |
|  | 75 to 84 | 31.4 (50) | - | - | 29.7 (76) | 30.7 (96.9) | 31.0 (98.9) | 27.7 (74) | 28.4 (90.8)* | 28.6 (94.1)* |
|  | 85 and above | 30.1 (30) | 32.6 (74.7) | - | 29.2 (63) | 30.4 (93.7) | 30.8 (97.6) | 27.5 (69) | 28.4 (90.3)* | 28.8 (95.4)* |
|  | Overall | 30.6 (37) | - | - | 29.5 (71) | 30.6 (96.6) | 31.0 (98.9) | 27.6 (71) | 28.5 (91.2)* | 28.8 (95.4)* |
| Pneumonia and Influenza | 15 to 64 | 30.9 (42) | 35.2 (99.6) | - | 29.7 (76) | 30.6 (95.9) | 31.0 (98.7) | 28.1 (85) | 29.2 (98.7) | 29.7 (99.9) |
|  | 15 to 74 | 31.4 (49) | - | - | 29.6 (74) | 30.7 (96.9) | 31.1 (99.2) | 27.7 (74) | 28.6 (94.0) | 28.9 (96.8) |
|  | 65 and above | 30.1 (29) | - | - | 29.3 (65) | 30.4 (94.1)* | 30.8 (97.7)* | 27.5 (70) | 28.4 (90.8)* | 28.6 (94.1)* |
|  | 65 to 74 | 31.4 (50) | - | - | 29.3 (65) | 30.8 (97.6) | 31.4 (99.8) | 27.5 (70) | 28.4 (90.9) | 28.7 (94.3) |
|  | 75 and above | 29.9 (27) | 32.6 (74.7) | - | 29.3 (64) | 30.4 (94.1)* | 30.7 (97.1)* | 27.5 (70) | 28.3 (88.3)* | 28.6 (94.1)* |
|  | 75 to 84 | 30.5 (35) | - | - | 29.5 (69) | 30.4 (94.1) | 30.7 (96.9) | 27.6 (72) | 28.3 (88.3) | 28.6 (94.0)* |
|  | 85 and above | 31.4 (50) | 32.8 (79.0)* | - | 29.1 (59) | 30.3 (92.5)* | 30.7 (97.1)* | 27.4 (68) | 28.3 (88.3)* | 28.6 (94.1)* |
|  | Overall | 30.2 (31) | - | - | 29.3 (65) | 30.4 (94.2)* | 30.8 (97.7)* | 27.5 (70) | 28.4 (90.8)* | 28.7 (94.5)* |

OT = optimal temperature; pct = temperature percentile; * = statistically significant, p≤0.05. Models adjusted for long-term trend (day of study), seasonality (day of year), day of week, holiday, rainfall, and PM2.5.

Maximum temperatures were non-significantly associated with only those 75 and above or 85 and above for mortality of all disease sub-groups (circulatory disease, respiratory, and pneumonia and influenza). Mean temperatures were also associated with all disease-specific sub-groups 65 and above, albeit non-significantly aside from pneumonia and influenza mortality. Minimum temperatures were not associated with circulatory mortality but were significantly associated with respiratory and pneumonia and influenza mortality aged 65 and above.

**Table A.7. Disease-specific hospital admission associations for maximum, mean, and minimum temperatures**

| **Diagnoses groups** | **Age groups** | **Maximum temp** | | | **Mean temp** | | | **Minimum temp** | | |
| --- | --- | --- | --- | --- | --- | --- | --- | --- | --- | --- |
|  |  | OT (pct) | RR 1.05 (pct) | RR 1.10 (pct) | OT (pct) | RR 1.05 (pct) | RR 1.10 (pct) | OT (pct) | RR 1.05 (pct) | RR 1.10 (pct) |
| Circulatory | 15 to 64 | 31.4 (50) | - | - | 28.1 (38) | - | - | 26.6 (50) | - | - |
|  | 15 to 74 | 31.4 (50) | - | - | 28.5 (47) | - | - | 26.6 (50) | - | - |
|  | 65 and above | 31.4 (50) | - | - | 28.7 (50) | - | - | 28.2 (86) | 29.9 (100.0) | - |
|  | 65 to 74 | 33.4 (90) | 35.9 (99.9) | - | 28.7 (50) | - | - | 26.6 (50) | - | - |
|  | 75 and above | 31.4 (50) | - | - | 28.7 (50) | - | - | 28.0 (83) | 29.3 (99.3)* | 29.9 (100.0)* |
|  | Overall | 31.4 (50) | - | - | 28.7 (50) | - | - | 28.5 (92) | - | - |
| Respiratory | 15 to 64 | 31.4 (50) | - | - | 30.0 (83) | 32.1 (99.9) | - | 27.9 (80) | 29.2 (98.5) | 29.8 (99.9) |
|  | 15 to 74 | 31.4 (50) | - | - | 30.0 (83) | - | - | 27.9 (79) | 29.4 (99.4) | - |
|  | 65 and above | 33.5 (92) | - | - | 30.2 (91) | - | - | 27.9 (79) | 30.0 (100.0) | - |
|  | 65 to 74 | 33.7 (93) | 36.3 (100.0) | - | 29.7 (76) | 31.0 (98.9) | 31.6 (99.8) | 27.8 (76) | 28.9 (96.8) | 29.4 (99.4) |
|  | 75 and above | 33.7 (93) | - | - | 30.1 (88) | - | - | 27.9 (79) | 29.8 (99.9) | - |
|  | Overall | 31.4 (50) | - | - | 28.7 (50) | - | - | 27.9 (80) | - | - |
| Pneumonia and Influenza | 15 to 64 | 31.4 (50) | - | - | 29.7 (75) | 30.7 (97.1) | 31.1 (99.1) | 27.8 (78) | 28.7 (94.6)* | 29.0 (97.6)* |
|  | 15 to 74 | 31.4 (50) | - | - | 29.7 (76) | 31.0 (98.7) | 31.5 (99.8) | 27.9 (79) | 28.8 (95.6)* | 29.2 (98.7)* |
|  | 65 and above | 31.4 (50) | - | - | 29.6 (74) | - | - | 27.7 (75) | 29.2 (98.7) | 29.8 (99.9)* |
|  | 65 to 74 | 33.7 (94) | 36.5 (100.0) | - | 29.9 (81) | 31.2 (99.3) | 31.8 (99.9) | 27.9 (79) | 28.7 (95.4) | 29.1 (97.9) |
|  | 75 and above | 31.4 (50) | - | - | 29.7 (76) | 32.1 (99.9) | - | 27.7 (75) | 29.2 (98.2) | 29.8 (99.9) |
|  | Overall | 31.4 (50) | - | - | 29.6 (74) | 31.8 (99.9) | - | 27.8 (76) | 29.1 (97.9)* | 29.6 (99.9)* |

OT = optimal temperature; pct = temperature percentile; * = statistically significant, p≤0.05. Models adjusted for long-term trend (day of study), seasonality (day of year), day of week, holiday, rainfall, PM2.5, and tropical cyclone.

Maximum temperatures were found non-significantly associated with only those 65-74 for all disease sub-groups. Mean temperatures were found non-significantly associated with pneumonia and influenza admissions. Minimum temperatures were significantly associated with circulatory disease admissions aged 75 and above and all ages for pneumonia and influenza admissions, but non-significantly associated for respiratory admissions. **Table A.8. Sensitivity analyses of non-cancer mortality associations for each temperature metric**

|  |  | **Maximum temp** | | | | | **Mean temp** | | | | | **Minimum temp** | | | | |
| --- | --- | --- | --- | --- | --- | --- | --- | --- | --- | --- | --- | --- | --- | --- | --- | --- |
|  |  | OT (pct) | RR 1.05 (pct) | RR 1.10 (pct) | R.sq | qAIC | OT (pct) | RR 1.05 (pct) | RR 1.10 (pct) | R.sq | qAIC | OT (pct) | RR 1.05 (pct) | RR 1.10 (pct) | R.sq | qAIC |
| 65 and above | **Original (adjusted for PM2.5)** | **30.70 (38.9)** | **-** | **-** | **0.193** | **10934.6** | **29.44 (68.9)** | **30.64 (96.1)*** | **31.08 (99.2)*** | **0.194** | **10930.5** | **27.57 (71.0)** | **28.65 (94.1)*** | **29.05 (97.7)*** | **0.195** | **10929** |
|  | Longer lag (0-10) | 30.9 (41) | - | - | 0.188 | 10945.5 | 29.5 (69) | 30.6 (95.7)* | 31.0 (98.6)* | 0.192 | 10934.8 | 27.6 (72) | 28.6 (94.0)* | 28.9 (96.8)* | 0.196 | 10925.4 |
|  | Relative Humidity | 30.0 (28) | - | - | 0.193 | 10933.6 | 29.4 (68) | 30.6 (96.4)* | 31.1 (99.1)* | 0.194 | 10930.2 | 27.6 (71) | 28.6 (94.1)* | 29.1 (97.6)* | 0.195 | 10930.2 |
|  | Wind | 30.20 (31.0) | - | - | 0.194 | 10806.3 | 29.37 (66.0) | 30.70 (97.1)* | 31.19 (99.4)* | 0.193 | 10806.4 | 27.50 (69.3) | 28.67 (94.2)* | 29.08 (97.9)* | 0.194 | 10805.6 |
|  | Month | 30.34 (33.1) | - | - | 0.191 | 10943.1 | 29.44 (68.9) | 30.68 (97.0)* | 31.14 (99.1)* | 0.192 | 10939.4 | 27.57 (71.0) | 28.68 (94.2)* | 29.09 (97.9)* | 0.192 | 10938.1 |
|  | Tropical cyclone | 30.70 (39.0) | - | - | 0.193 | 10935.5 | 29.50 (71.0) | 30.65 (97.0)* | 31.09 (99.2)* | 0.194 | 10931 | 27.60 (72.2) | 28.65 (94.1)* | 29.04 (97.7)* | 0.195 | 10928.7 |
|  | PM10 | 30.70 (39.4) | - | - | 0.193 | 10934 | 29.44 (68.9) | 30.66 (97.2)* | 31.10 (99.2)* | 0.194 | 10930.4 | 27.57 (71.0) | 28.66 (95.3)* | 29.06 (98.0)* | 0.195 | 10928.8 |
|  | NO2 | 30.79 (39.5) | - | - | 0.192 | 10935.1 | 29.50 (71.5) | 30.62 (96.0)* | 31.05 (99.0)* | 0.194 | 10930.1 | 27.60 (72.1) | 28.65 (94.1)* | 29.03 (97.7)* | 0.195 | 10929.3 |
|  | Nox | 30.80 (40.8) | - | - | 0.193 | 10933.1 | 29.50 (71.5) | 30.63 (96.0)* | 31.06 (99.0)* | 0.195 | 10928 | 27.60 (72.1) | 28.65 (94.1)* | 29.04 (97.7)* | 0.195 | 10927.7 |
|  | O3 | 30.79 (39.5) | - | - | 0.193 | 10933 | 29.44 (68.9) | 30.67 (97.2)* | 31.12 (99.2)* | 0.194 | 10929.6 | 27.57 (71.0) | 28.66 (95.3)* | 29.06 (98.0)* | 0.195 | 10927.7 |
|  | SO2 | 30.50 (36.0) | - | - | 0.195 | 10930.2 | 29.44 (68.9) | 30.62 (96.0)* | 31.07 (99.0)* | 0.197 | 10925.7 | 27.57 (71.0) | 28.65 (94.1)* | 29.04 (97.7)* | 0.197 | 10925.8 |
|  | CO | 30.50 (35.4) | - | - | 0.194 | 10932.9 | 29.44 (68.9) | 30.63 (96.0)* | 31.07 (99.0)* | 0.195 | 10927.7 | 27.57 (71.0) | 28.66 (95.3)* | 29.05 (98.0)* | 0.196 | 10927.4 |
| Overall | **Original (adjusted for PM2.5)** | **30.90 (42.1)** | **-** | **-** | **0.184** | **11104.5** | **29.44 (68.9)** | **30.71 (96.9)*** | **31.19 (99.3)*** | **0.185** | **11103.8** | **27.50 (70.5)** | **28.68 (94.9)*** | **29.11 (97.8)*** | **0.185** | **11104.5** |
|  | Longer lag (0-10) | 30.8 (40) | - | - | 0.176 | 11122.9 | 29.4 (68) | 30.6 (95.7)* | 31.0 (98.9)* | 0.181 | 11111.6 | 27.5 (70) | 28.6 (94.0)* | 28.9 (96.8)* | 0.186 | 11101.7 |
|  | Relative Humidity | 30.3 (32) | - | - | 0.185 | 11103.5 | 29.4 (66) | 30.7 (97.2)* | 31.2 (99.3)* | 0.185 | 11103.1 | 27.5 (69) | 28.7 (94.5)* | 29.1 (98.1)* | 0.185 | 11105.7 |
|  | Wind | 30.50 (35.3) | - | - | 0.186 | 10975.8 | 29.37 (66.0) | 30.76 (97.9)* | 31.30 (99.6)* | 0.185 | 10978.1 | 27.40 (68.2) | 28.68 (94.9)* | 29.13 (98.2)* | 0.185 | 10979 |
|  | Month | 30.40 (34.7) | - | - | 0.183 | 11112.1 | 29.40 (67.4) | 30.72 (96.9)* | 31.22 (99.4)* | 0.183 | 11111.2 | 27.49 (69.0) | 28.68 (94.9)* | 29.12 (98.2)* | 0.184 | 11110.7 |
|  | Tropical cyclone | 30.90 (42.1) | - | - | 0.184 | 11106 | 29.44 (68.9) | 30.71 (96.9)* | 31.19 (99.3)* | 0.185 | 11104.8 | 27.50 (70.7) | 28.68 (94.9)* | 29.10 (98.0)* | 0.185 | 11104.6 |
|  | PM10 | 30.90 (42.2) | - | - | 0.185 | 11104.2 | 29.44 (68.9) | 30.71 (97.1)* | 31.20 (99.4)* | 0.185 | 11103.8 | 27.49 (69.0) | 28.68 (94.7)* | 29.11 (98.1)* | 0.185 | 11104.6 |
|  | NO2 | 30.90 (41.1) | - | - | 0.184 | 11104.7 | 29.44 (68.9) | 30.69 (97.2)* | 31.16 (99.4)* | 0.185 | 11103.1 | 27.50 (69.3) | 28.67 (94.7)* | 29.09 (97.9)* | 0.185 | 11104.6 |
|  | Nox | 31.00 (43.4) | - | - | 0.185 | 11103 | 29.44 (68.9) | 30.70 (96.9)* | 31.18 (99.4)* | 0.186 | 11101.2 | 27.50 (69.3) | 28.68 (94.7)* | 29.10 (98.0)* | 0.185 | 11103.1 |
|  | O3 | 30.80 (40.6) | - | - | 0.185 | 11103.3 | 29.44 (68.9) | 30.75 (97.7)* | 31.25 (99.3)* | 0.185 | 11102.9 | 27.49 (69.0) | 28.68 (94.7)* | 29.12 (98.2)* | 0.185 | 11103 |
|  | SO2 | 30.79 (39.5) | - | - | 0.187 | 11101.2 | 29.37 (66.0) | 30.69 (97.2)* | 31.18 (99.4)* | 0.187 | 11099.9 | 27.49 (69.0) | 28.68 (94.7)* | 29.11 (98.1)* | 0.186 | 11101.8 |
|  | CO | 30.70 (38.9) | - | - | 0.186 | 11101.5 | 29.40 (67.3) | 30.69 (97.2)* | 31.16 (99.4)* | 0.187 | 11099.2 | 27.49 (69.0) | 28.68 (94.7)* | 29.12 (98.2)* | 0.186 | 11101 |

OT = optimal temperature; pct = temperature percentile; * = statistically significant, p≤0.05. Models adjusted for long-term trend (day of study), seasonality (day of year), day of week, holiday, rainfall, and PM2.5. Models adjusted for wind have less observations due to missing data (n = 1523, vs original n = 1530).

**Table A.9. Sensitivity analyses of non-cancer hospital admission associations for each temperature metric**

|  |  | **Maximum temp** | | | | | **Mean temp** | | | | | **Minimum temp** | | | | |
| --- | --- | --- | --- | --- | --- | --- | --- | --- | --- | --- | --- | --- | --- | --- | --- | --- |
|  |  | OT (pct) | RR 1.05 (pct) | RR 1.10 (pct) | R.sq | qAIC | OT (pct) | RR 1.05 (pct) | RR 1.10 (pct) | R.sq | qAIC | OT (pct) | RR 1.05 (pct) | RR 1.10 (pct) | R.sq | qAIC |
| 65 and above | **Original (adjusted for PM2.5)** | **31.40 (50)** | **-** | **-** | **0.823** | **16658.2** | **29.90 (82.2)** | **-** | **-** | **0.826** | **16604.5** | **27.90 (79.9)** | **29.83 (99.9)*** | **-** | **0.823** | **16661** |
|  | Longer lag (0-10) | 31.4 (50) | - | - | 0.823 | 16677.1 | 30.2 (89) | - | - | 0.826 | 16610.8 | 28.0 (81) | 29.6 (99.8)* | - | 0.822 | 16697.3 |
|  | Relative Humidity | 31.4 (50) | - | - | 0.824 | 16642.2 | 29.7 (75) | - | - | 0.827 | 16587.1 | 27.9 (80) | 29.9 (100.0)* | - | 0.824 | 16652.2 |
|  | Wind | 31.40 (50) | - | - | 0.826 | 16416.6 | 30.00 (84.2) | - | - | 0.828 | 16355.3 | 27.90 (79.9) | 29.90 (100.0)* | - | 0.826 | 16401.7 |
|  | Month | 31.40 (50) | - | - | 0.823 | 16666.3 | 29.91 (82.4) | - | - | 0.825 | 16612.7 | 27.90 (79.9) | 29.79 (99.9)* | - | 0.823 | 16666.5 |
|  | PM10 | 31.40 (50) | - | - | 0.823 | 16658.3 | 29.91 (82.4) | - | - | 0.826 | 16605.3 | 27.90 (78.3) | 29.87 (100.0)* | - | 0.823 | 16661.3 |
|  | NO2 | 31.40 (50) | - | - | 0.823 | 16654.4 | 29.87 (80.1) | - | - | 0.826 | 16599.8 | 27.90 (80.6) | 29.81 (99.9)* | - | 0.823 | 16654.9 |
|  | Nox | 31.40 (50) | - | - | 0.823 | 16657.1 | 29.87 (80.1) | - | - | 0.826 | 16605.2 | 27.90 (78.3) | 29.77 (99.9)* | - | 0.823 | 16654.5 |
|  | O3 | 31.40 (50) | - | - | 0.823 | 16661.4 | 29.87 (80.1) | - | - | 0.826 | 16607.5 | 27.90 (78.3) | 29.81 (99.9)* | - | 0.823 | 16664 |
|  | SO2 | 31.40 (50) | - | - | 0.824 | 16647.4 | 29.87 (80.1) | - | - | 0.826 | 16597.4 | 27.90 (78.3) | 29.81 (99.9)* | - | 0.823 | 16654.5 |
|  | CO | 31.40 (50) | - | - | 0.826 | 16609.9 | 29.87 (80.1) | - | - | 0.829 | 16547 | 27.90 (79.1) | 29.99 (100.0)* | - | 0.826 | 16605.8 |
| Overall | **Original (adjusted for PM2.5)** | **31.40 (50)** | **-** | **-** | **0.858** | **18282.5** | **29.70 (74.3)** | **-** | **-** | **0.859** | **18241.7** | **27.80 (77.2)** | **-** | **-** | **0.859** | **18264.5** |
|  | Longer lag (0-10) | 31.4 (50) | - | - | 0.858 | 18358 | 30.3 (92) | - | - | 0.859 | 18315.4 | 28.0 (81) | 29.7 (99.9)* | - | 0.858 | 18350.6 |
|  | Relative Humidity | 31.4 (50) | - | - | 0.858 | 18326.4 | 28.7 (50) | - | - | 0.86 | 18281.1 | 27.8 (78) | - | - | 0.859 | 18311.3 |
|  | Wind | 31.40 (50) | - | - | 0.86 | 17999.3 | 29.73 (76.5) | - | - | 0.862 | 17942.4 | 27.80 (77.2) | - | - | 0.862 | 17953.5 |
|  | Month | 31.40 (50) | - | - | 0.858 | 18300.8 | 29.73 (76.5) | - | - | 0.859 | 18261.2 | 27.80 (77.2) | - | - | 0.858 | 18280.6 |
|  | PM10 | 31.40 (50) | - | - | 0.858 | 18281.9 | 29.70 (75.3) | - | - | 0.859 | 18240.8 | 27.80 (75.8) | - | - | 0.859 | 18263.8 |
|  | NO2 | 31.40 (50) | - | - | 0.858 | 18275.9 | 29.50 (69.2) | - | - | 0.86 | 18229.3 | 27.80 (76.7) | - | - | 0.859 | 18251.4 |
|  | Nox | 31.40 (50) | - | - | 0.858 | 18274.4 | 29.64 (74.1) | - | - | 0.86 | 18234.2 | 27.80 (75.8) | - | - | 0.859 | 18249 |
|  | O3 | 31.40 (50) | - | - | 0.858 | 18287.4 | 29.60 (73.7) | - | - | 0.859 | 18245 | 27.80 (75.8) | - | - | 0.859 | 18269.8 |
|  | SO2 | 31.40 (50) | - | - | 0.859 | 18268.7 | 29.70 (75.3) | - | - | 0.86 | 18233.6 | 27.80 (75.8) | - | - | 0.859 | 18256.9 |
|  | CO | 31.40 (50) | - | - | 0.86 | 18238.3 | 28.70 (50) | - | - | 0.861 | 18183.2 | 27.80 (76.7) | - | - | 0.861 | 18211.8 |

OT = optimal temperature; pct = temperature percentile; * = statistically significant, p≤0.05. Models adjusted for long-term trend (day of study), seasonality (day of year), day of week, holiday, rainfall, PM2.5, and tropical cyclone. Models adjusted for wind have less observations due to missing data (n = 1523, vs original n = 1530).
